# Reimplantable Microdrive for Long-Term Chronic Extracellular Recordings in Freely Moving Rats

**DOI:** 10.3389/fnins.2019.00128

**Published:** 2019-02-21

**Authors:** Leopoldo Emmanuel Polo-Castillo, Miguel Villavicencio, Leticia Ramírez-Lugo, Elizabeth Illescas-Huerta, Mario Gil Moreno, Leopoldo Ruiz-Huerta, Ranier Gutierrez, Francisco Sotres-Bayon, Alberto Caballero-Ruiz

**Affiliations:** ^1^Instituto de Ciencias Aplicadas y Tecnología, Universidad Nacional Autónoma de México, Mexico City, Mexico; ^2^National Laboratory for Additive and Digital Manufacturing, Mexico City, Mexico; ^3^Laboratory of Neurobiology of Appetite, Department of Pharmacology, Centro de Investigación y de Estudios Avanzados, Mexico City, Mexico; ^4^Instituto de Fisiología Celular - Neurociencias, Universidad Nacional Autónoma de México, Mexico City, Mexico

**Keywords:** microdrive, extracellular chronic recording, reimplantable microdrive, electrophysiology, additive manufacturing

## Abstract

Extracellular recordings of electrical activity in freely moving rats are fundamental to understand brain function in health and disease. Such recordings require a small-size, lightweight device that includes movable electrodes (microdrive) to record either a new set of neurons every day or the same set of neurons over time. Ideally, microdrives should be easy to implant, allowing precise and smooth displacement of electrodes. The main caveat of most commercially available microdrives is their relatively short half-life span, in average ranging from weeks to a month. For most experiments, recording days–weeks is sufficient, but when the experiment depends on training animals for several months, it is crucial to develop new approaches. Here, we present a low-cost, reusable, and reimplantable device design as a solution to extend chronic recordings to long-term. This device is composed of a baseplate that is permanently fixed to the rodent’s skull, as well as a reusable and replaceable microdrive that can be attached and detached from the baseplate, allowing its implantation and reimplantation. Reimplanting this microdrive is particularly convenient when no clear neuronal signal is present, or when the signal gradually decays across days. Our microdrive incorporates a mechanism for moving a 16 tungsten-wire bundle within a small (∼15 mm^3^) lightweight device (∼4 g). We present details of the design, manufacturing, and assembly processes. As a proof of concept, we show that recordings of the nucleus accumbens core (NAcc) in a freely behaving rat are stable over a month. Additionally, during a lever-press task, we found, as expected, that NAc single-unit activity was associated with rewarded lever presses. Furthermore, we also show that NAc shell (NAcSh) responses evoked by freely licking for sucrose, consistent with our previously published results, were conserved from a first implant to a second microdrive reimplant in the same rat, notably showing reimplantation is possible without overtly affecting the functional responses of the area of interest. In sum, here we present a novel microdrive design (low-cost, small size, and light weight) that can be used for long-term chronic recordings and reimplanted in freely behaving rats.

## Introduction

Electrophysiological recording of brain activity is a valuable technique to understand the neural correlates of behavior in health and disease (Esmaeili and Grace, 2013). Recordings of electrical neural activity in rodents provide useful insights into how the brain represents information about the internal and external world (Nicolelis et al., 1995; Buzsáki, 2004; Hasegawa et al., 2015), uncovering the neuronal correlates from sensory and perceptual processing to decision making (Feierstein et al., 2006; Fonseca et al., 2018). In general, the methods in electrophysiology can be divided into intracellular and extracellular recordings. Unlike the former, the latter is widely used in freely moving animals, which allows stable recordings for several minutes or even hours (Ribeiro et al., 2004; Jovalekic et al., 2017). However, the loss of signal becomes a fundamental problem, especially, in studies involving either extensive behavioral training (spanning from weeks to months) or in animal models characterizing the development of chronic human diseases (Vlamings et al., 2012; Ellens and Leventhal, 2013; Sesia et al., 2013; Saddoris et al., 2016). To circumvent this issue, it is needed a reimplantable microdrive to record, the same animal, over an extended period.

Although it is not fully understood why electrical signals degrade along time, it has been speculated that the loss of signal could be caused by tiny vibrations produced by the animal’s movement or by biological reasons such as tissue reaction surrounding the electrode, resulting in neuronal migration, neuronal death, or glial encapsulation of the recording tips (Muthuswamy et al., 2005, 2011; Branchaud et al., 2006). Also, researchers face the problem of oversampling the same neuropil over days, which reduces the biological replicates within a dataset. In this regard, previous microdrives were developed to move the wires and to record from a new set of neurons every day (Gutierrez et al., 2010) or record from the same neurons for a few days (Sotres-Bayon et al., 2012). In any case, most commonly movable electrodes are permanently implanted and cemented to the skull which precludes reimplantation in the same subject and thus increased the number of experimental animals needed (Fitts, 2011).

Multiple designs have been used for building microdrives. Manually movable microdrives have been used in the past decades with limited success. Morrow (1980) reported a manual mechanism which is based on four parts with a mounting sleeve, a carrier tube and an electrode drive screw that moves the electrode. Later, O’Keefe and Recce (1993) reported the use of a microdrive with a mechanism that avoids the rotation of the cannula or electrodes. Gothard et al. (1996) developed a conic and bulky microdrive able to move 14 tetrodes independently. Looking for a low-cost, low-weight, and easy-fabrication device, the “Scribe microdrive” was presented by Bilkey and Muir (1999). It is composed of the parts of a pen, a guide cannula and a screw, whose motion is transmitted using a contact point between the screw and the pen nib, allowing only forward displacement. Lansink et al. (2007) reported the development of a multi-electrode microdrive for chronic recording in two brain areas simultaneously, based on a microdrive presented by Gothard et al. (1996). This system allows reusing some parts of the microdrive. More recently, Chang et al. (2013) developed a home-made microdrive consisting of two joined brass guides. This microdrive uses four tetrodes displaced by a screw, and an electronic interface board (EIB-18, Neuralynx) for proper connections, which resulted in stable single-unit recordings for several weeks. Also, the same manuscript shows the steps for assembling a commercially available microdrive (Versa Drive 4, Neuralynx) based on a Mill-Max commercial connector and containing four drive screws (Chang et al., 2013).

The main disadvantage of manually driven microdrives is the limited precision to move electrodes and the stress to which the animal is subjected when lowering them. To solve this problem, several automatic movable microdrives have been developed. Fee and Leonardo (2001) introduced a microdrive consisting of a brushless dc micromotor. In 2007, a microdrive based on a hydraulic system was presented. This device can move 22 wires independently along 4 mm in intervals of 50 μm (Sato et al., 2007). Yamamoto and Wilson (2008) reported a motorized system with 21 drivers, each composed of five parts: a dc brushless servomotor with planetary gearbox, a threaded rod, a slotted PEEK tube, a shuttle lock, and a shuttle. A microdrive for mice containing a piezo-motor (TULA 35, Piezoelectric Technology Co., Ltd.) with 1 μm accuracy was presented by Yang et al. (2011). This motorized microdrive comprises 13 components. Another lightweight microdrive that uses a dc micromotor with threaded output was developed in 2012 by Otchy and Ölveczky, which consists of seven major components. In 2014, Caballero-Ruiz et al. (2014) proposed a high-precision displacement system based on a piezo motor (SQL-RV-1.8, New Scale Technologies Inc.) and a Hall-effect sensor (Allegro A1324 MicroSystems, Inc.) comprising six assembly parts and three more for the closed-loop positioning system. In 2017, Jovalekic et al. (2017) developed another micropositioning system consisting of a piezo motor (SQL-RV-1.8, New Scale Technologies Inc.) and a Hall-effect sensor (NSE-5310, New Scale Technologies, Inc.). These electrodes could be replaced, increasing the yield of neurons recorded. However, to further increase the number of neurons recorded, there is a need for a reimplantable microdrive for chronic recordings in freely behaving animals. This is particularly relevant in experiments in which much time and effort are devoted to training animals in tasks where reusing and reimplanting microdrives would increase the gathering of neural data.

Among the many microdrives developed so far, the power screw mechanism is the most commonly used for electrode positioning, whether it is a manual or automated mechanism. Most of the automated ones, in addition to the screw, use geared brushless dc micromotors, reaching resolutions about 1 μm. As suggested by Sato et al. (2007), and in spite of the high resolutions achieved by this kind of mechanisms, the backlash present in the gears and leadscrews introduces a significant amount of imprecision which could cause lateral deviations along the vertical displacement (as it can be seen in the work carried out by Chang et al., 2013) and, even more, the micro motion between the electrode and the surrounding tissue, due to the natural movements (i.e., heartbeat and respiration) of the animal, could results in loss of signal (Muthuswamy et al., 2011). To address this problem, Sato et al. (2007) put forward a hydraulic system in which electrode positioning can be estimated accurately with a pre-pressurized system. Although the hydraulic microdrive can precisely displace the electrodes, it is too heavy and bulky. From another standpoint, electrode motion can be performed using piezoelectric motors. Unlike electromagnetic devices, the piezoelectric ones are electromagnetic noise-free, and their efficiency is insensitive to size (Heywang et al., 2008). Besides considering low weight, low cost, small dimensions, and high precision as important requirements for the development of microdrives, it is also important to consider the half-life of electrodes. Long-term experiments require a reliable microdrive to record neural signals over a long period able to overcome biotic and abiotic factors that could affect impedance(Sankar et al., 2014).

In this work, we present the development of a reusable and reimplantable microdrive that increases the success of electrophysiological studies in freely moving rats. Such a microdrive is based on a smooth displacement mechanism and a reimplantable system. It also accomplishes the low weight, low cost, small dimension, and high precision requirements mentioned above. The proposed mechanism prevents lateral motion along the electrode stroke, which results in stable recordings. The motion can be manually or automated performed, depending on the preference of the user. Moreover, a baseplate is fixed to the skull without using dental acrylic, and the microdrive is attached to the baseplate. Thus, it can be easily replaced. The efficiency of the microdrive was demonstrated using electrophysiological experiments in freely behaving rats. First, we characterized the stability of our microdrive. We found stable responses in a single neuron recorded in the nucleus accumbens core (NAcc) while a rat pressed a lever to obtain food. Then, in a separate rat, the spontaneous activity of two neurons was successfully recorded for over a month (40 days). Having demonstrated the stability of the microdrive, we evaluated if our microdrive could be reimplanted, in the same rat, without overtly damaging the functional activity of the brain region of interest. To this end, single-unit recordings from the NAc shell (NAcSh) were performed while rats licked for sucrose. In the same animals, we found that the signal quality between the first electrode implant and the second one was not statistically different, indicating that the proposed reimplantable microdrive can potentially extend the half-life of the electrophysiological experiments. Also, it revealed that reimplantation did not impair the normal function of the recorded brain region since our data replicated a well-known function of the NAcSh. That is, the majority of NAcSh neurons were either Lick-Inactive whereas the minority were Lick-Active during consummatory behavior, in the same proportion than previous observations (Krause et al., 2010; Tellez et al., 2012; Villavicencio et al., 2018). In sum, we demonstrated that the proposed reimplantable microdrive could be a reliable solution to extend chronic extracellular neuronal recording in freely behaving rats.

## Materials and Methods

In this section, the design, fabrication, assembly, and implementation of a reimplantable microdrive to smoothly displace electrode bundles for extracellular recordings is presented. Such a microdrive is based under the following requirements: small-size less than 15 mm × 15 mm × 15 mm; weight less than 5 g; mechanical stability; able to move a bundle of 16 microelectrodes; able to be actuated manually or by motor (for manual actuating, using a resolution between 50 and 100 μm; while for motorized actuating, employing a resolution less than 10 μm); able to be reimplanted and easy to be installed and reused.

### Microdrive Design

**FIGURE 1:**
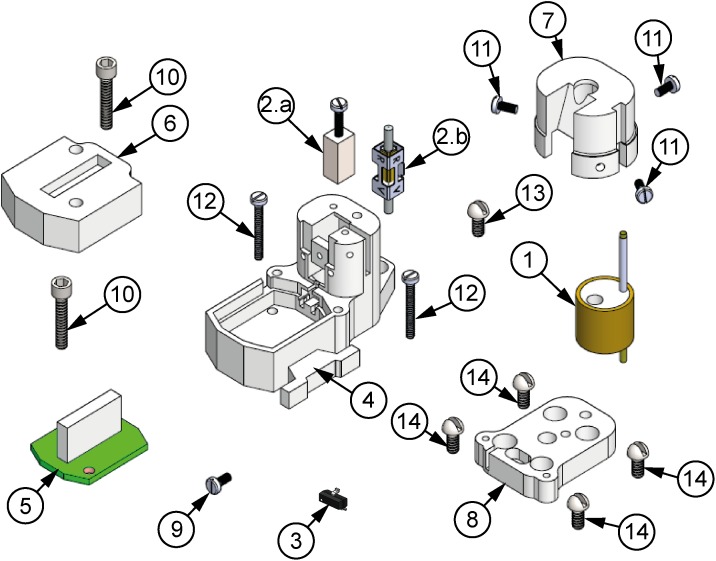
Exploded view of the microdrive proposed. **(1)** Electrode shuttle system, **(2a)** nut-screw linear motion system, **(2b)** piezoelectric motor, **(3)** Hall-effect sensor, **(4)** housing element, **(5)** EIB-18, **(6)** EIB-18 cover, **(7)** electrode cover, **(8)** baseplate, **(9)** fixing screw of the motor or nut, **(10)** EIB cover-housing fixing screw (×2), **(11)** electrode cover-housing fixing screws (×3), **(12)** posterior housing-baseplate fixing screws (×2), **(13)** anterior housing-baseplate fixing screw, **(14)** baseplate-skull fixing screws (×4).

Figure 1 depicts the exploded view of the proposed microdrive, which has eight overall components: a micropositioning mechanism, composed of an electrode shuttle system (1) able to move the electrodes by means of either a nut-screw linear motion system (2a), for manual configuration, or a piezoelectric motor (SQL-RV-1.8; NewScale Tech.) (2b), for motorized configuration; in order to obtain precise displacements, a closed-loop position control system is used based on a Hall-effect sensor (Allegro A1324 MicroSystems, Inc.) (3). Inside the housing (4), the mechanism, the actuator and the gold-plated electronic interface board (EIB; 5) are located. Also, the design has two covers, one that is designated to protect the EIB (6) and the other one to protect the electrodes (7). Finally, the entire assembly lies on top of the baseplate (8), which is the only piece permanently fixed on the skull of the rat.

**FIGURE 2:**
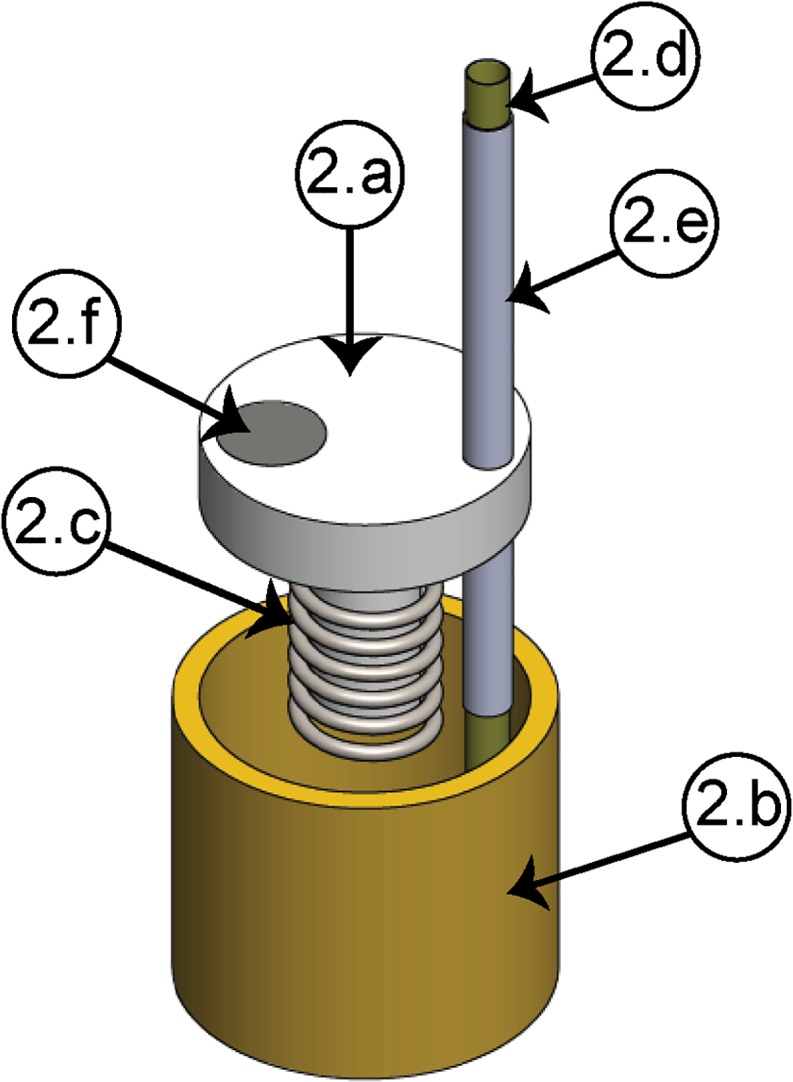
Electrode shuttle system. **(2a)** Piston, **(2b)** bushing, **(2c)** compression spring, **(2d)** polyimide tube, **(2e)** cannula guide, **(2f)** magnet.

In order to fulfill the requirements for high resolution and mechanical stability, the linear motion is generated by either a nut-screw system [Figure 1(2a)], for the manual configuration, or by a piezoelectric actuator [Figure 1(2b)], for the motorized configuration, which is coupled to an electrode shuttle system (see Figure 2). The electrode shuttle system is composed of an element acting as a piston [Figure 2 (2a)] through a bushing (2b) in order to constrain the displacement. A compression spring (2c) introduces a pre-load between the piston and the nut-screw system or the piezoelectric actuator, warranting cero backlash. In this way, the mechanism exhibits high stiffness, load capacity and resistance to shock and vibration. The piston works as a shuttle, in which a stainless-steel guide cannula (2e) with a polyimide tube (2d) concentrically arranged is fixed. To prevent short circuits between the microelectrodes and the cannula, the polyamide tube is used, and the cannula guide improves the shuttle system alignment. For the motorized configuration, a magnet (2f), sensed by the Hall effect sensor, is employed.

**FIGURE 3:**
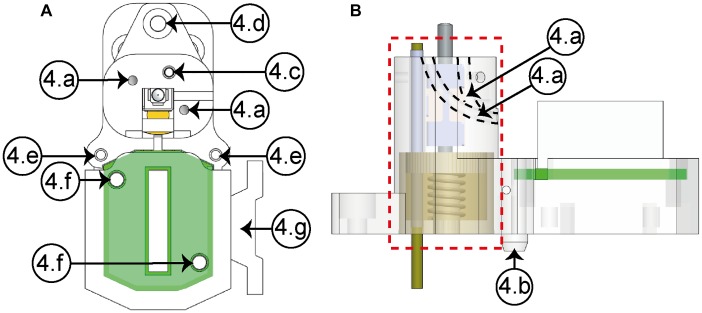
Housing element (**A**, upper view; **B**, lateral view). **(4a)** Conduits (×2), **(4b)** guide wedge, **(4c)** cannula hole, **(4d)** anterior fastening hole, **(4e)** posterior fastening holes (×2), **(4f)** EIB’s fastening holes (×2), **(4g)** headstage clamping element.

The housing element (Figure 3) and the two covers shape the casing. Inside the first one, the micropositioning mechanism is located, as it can be seen in the lateral view (dashed line, red square). In the housing element, two internal conduits [Figure 3 (4a)] permit guiding the electrodes to the EIB, protecting them from damages caused by the natural movements of the animal. In this way, the electrodes can be led from the top to the bottom of the structure in order to be finally cut and connected to the EIB. The clamping element (4g) permits a stable attachment between the connector and the headstage using an elastic rubber band. The EIB cover is fastened to the housing element using two screws through the holes (4f). Using three screws, the electrode cover is fixed to the housing, completing in this way the protection of the electrodes from the movements of the animal.

**FIGURE 4:**
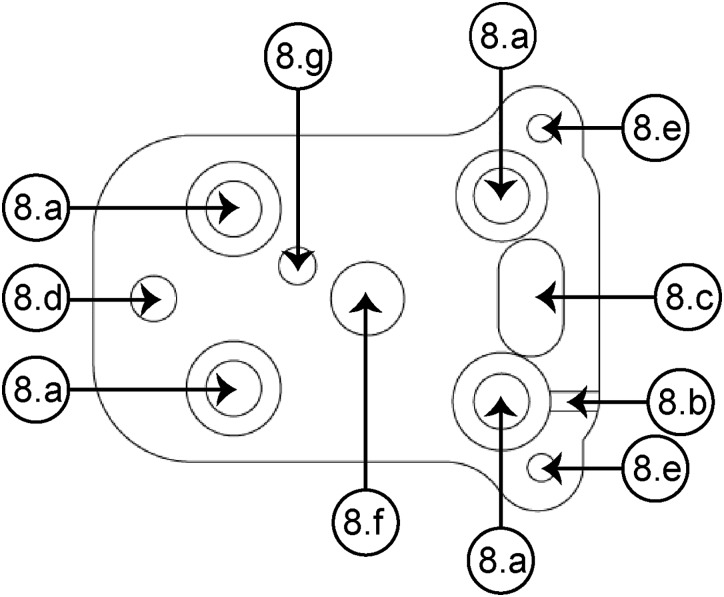
Superior view of the baseplate. **(8a)** Baseplate-skull fastening hole (×4), **(8b)** groove for the ground wire, **(8c)** slotted guide groove, **(8d)** anterior fastening hole, **(8e)** posterior fastening hole (×2), **(8f)** piston blind hole, **(8g)** recording site (for the right NAcSh).

The baseplate (see Figure 4) is the element that facilitates the mounting on the skull of the rat. Such a baseplate can be easily designed for a desired position. It allows the device to be replaced in case of loss of signal, increasing the experiment time without the necessity of a second surgery. Through the fastening holes [Figure 4 (8a)], four screws have the function of fixing the baseplate to the skull. A groove (8b) allows joining the ground wire to the proper pin of the EIB. The alignment between the slotted guide groove (8c) and the guide wedge [Figure 3(4b)] makes it easier to assemble the housing element on the baseplate; also, three screws permit fixing both elements by means of an anterior fastening hole (8d) and two posterior fastening holes (8e). Also, the baseplate includes a hole (8f) through which the piston travels in a vertical path together with the bundle of electrodes in the recording site hole (8g). A microdrive design that includes such baseplate provides the opportunity to easily replace the device if there is an error during surgery.

### Microdrive Fabrication and Assembly

To develop the microdrive presented, manufacturing processes criteria such as the shape of the components, type of the required materials as well as resolution and cost were considered. For the micropositioning mechanism, high resolution is a critical requirement, hence CNC manufacturing was used; through the milling process, the bushing and the piston were manufactured: the bushing was made of brass, with overall dimensions of 6 mm in internal diameter, 7 mm in external diameter, 6 mm in height and a weight of 0.29 g; while the piston was made of acrylic and has overall sizes of 2 mm in the inner diameter, 6 mm in the outer diameter, 6 mm in height and a weight of 0.069 g. The baseplate (weight: 0.52 g), the housing element (weight: 1.42 g), the covers (electrode cover weight: 0.42 g and EIB cover weight: 0.35094 g), and the nut (weight: 0.045 g) were manufactured using an additive manufacturing process (material jetting) because of the complexity of their shapes; the equipment employed was a Connex 3 by Stratasys, and the material used was VeroClear resin (RGD810). This technology has the advantage of reducing manufacturing costs when the production batch increases. The other components are commercial. The compression spring used (by Industrial Springs Corp.) is made of stainless steel and has a mean diameter of 2 mm, a free length of 5 mm and a weight of 0.01 g. The polyimide tube (Polyimide 0.0135 ID by Neuralynx) has a length of 18 mm and weighs only 0.002 g, while the guide cannula (20G by Lanceta HG) has a length of 13.5 mm and weighs 0.37 g. The magnet (R063-063 by Amazing Magnets LLC) weights 0.023 g and has overall sizes of 1.59 mm diameter and 1.59 mm length. The Hall-effect sensor (A1324LLHLT-T by Allegro MicroSystems) has overall sizes of 2 × 3 × 1 mm and weighs 0.015 g. In order to avoid rust problems, stainless steel screws were used which, according to their application on the microdrive design, can be classified into three types: power screw, housing fixing screws and skull fixing screws. The first one (#21475 by MetricScrews) is M1-0.25 × 10 mm and weighs 0.045 g. The second one is composed of three different screws: four pieces of M1-0.25 × 3 mm (#20694 by MetricScrews) with a weight of 0.07760 g, two pieces of M1-0.25 × 8 mm (#21474 by MetricScrews) screws with a weight of 0.068 g and one 0–80 × 1/8 (by Plastics One Inc.) screw with a weight of 0.058 g. Finally, the skull fixing screws consist of four 0–80 × 1/8” screws with a weight of 0.23332 g. For the automated microdrive, a piezoelectric SQL-RV-1.8 actuator by New Scale Technologies is used, such an actuator has overall sizes of 2.8 × 2.8 × 6 mm and a weight of 0.16 g.

**FIGURE 5:**
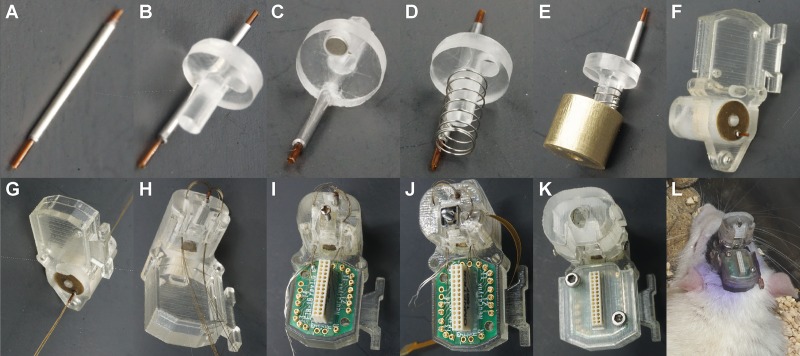
Reimplantable microdrive assembly. **(A)** Polyimide tube in the guide cannula. **(B)** Guide cannula fixed to the piston. **(C)** A magnet placed in the piston. **(D)** Compression spring placed concentrically to the piston rod. **(E)** Piston placed concentrically and aligned to the bushing. **(F)** Electrode shuttle system assembled with the housing element. **(G)** Electrodes introduced through the polyimide tube. **(H)** Electrodes introduced through the conduits. **(I)** Nut-screw and EIB placed in the housing element. **(J)** Piezoelectric actuator placed in the housing element for the automated configuration. **(K)** Final assembly with the two covers and the housing fixing screws. **(L)** Illustrative picture of a rat implanted with a reimplantable microdrive.

The complete assembly can be divided into three stages: the first one is related with the assembly of the micro positioning system; the second one involves the insertion and connection of the electrodes, and the last one is concerned with the placement of the protection covers with the appropriate fixing screws. The complete process can be seen in Figure 5. As a first step, the electrode shuttle system is assembled by fixing the polyimide tube and cannula (Figure 5A) to the piston (Figure 5B), the polyimide tube projects 2 mm from the lower face of the baseplate. Then, the magnet is placed in the piston hole (Figure 5C) and the compression spring is placed concentrically to the piston rod (Figure 5D); after that, all these parts are put concentrically in the bushing (Figure 5E), which in turn is placed in the housing element (Figure 5F). After this, the nut-screw or the piezoelectric actuator can be placed and fixed with a screw. This stage takes about 10 min. The second stage consists in introducing the electrodes through the polyimide tube (Figure 5G) and the conduits (Figure 5H). After this, the electrodes are cut 7.5 mm from the lower face of the baseplate for the NAcSh, and 6 mm for the NAcc in order to reach the region of interest (see Supplementary Figure S1); next, the EIB is placed in the housing element; and finally, the electrodes are connected to the EIB by means of gold-plated pins (Figure 5I). Figure 5J shows the assembly with the piezoelectric actuator. It is important to handle the electrodes carefully, making sure they do not bend. This task takes around 30 min. The last stage consists in protecting the EIB and the exposed part of the electrodes by fixing the covers with the housing element (Figure 5K). At the end of this stage, the final assembly time is less than 70 min. Including the weight of the baseplate, the total weight of the reimplantable microdrive is about 4.08 g when the nut-screw is used, and 4.15 g if the piezoelectric motor is implemented. In both configurations, its overall dimensions are 27.73 × 15.95 × 21.50 mm. Finally, before the implantation, the electrodes can be gold plated and then the device is ready. Supplementary Table S2 depicts the cost of all components to build one microdrive. In the case of the automated microdrive, the total cost of the parts is 280.2 USD. Considering that the actuator and the EIB are reusable, the total cost of each microdrive is approximately 53 USD (and it could be cheaper by producing a larger batch). All STL files are provided in the Supplementary Data Sheet S1.

#### Gold Plating of Electrodes

The impedance of the electrodes was controlled by electroplating the tip of the wires. Electroplating prevents impedance damping and improves the signal-to-noise ratio (Ferguson et al., 2009). Before implantation (and reimplantation), the 16 tungsten wires with 35 μm in diameter were electro-plated with a gold plating solution and the recommended Neuralynx stepped protocol (Supplementary Table S1) using a nanoZ^TM^ multi-electrode impedance tester (Plexon, Dallas, TX, United States). Impedances were reduced to 70 kΩ.

### Microdrive in Freely Behaving Rats

The proposed microdrive was tested in freely behaving animals. Testing involved two separate experiments in different laboratories. In both laboratories, the activity in the nucleus accumbens (NAc) of the ventral striatum was recorded, a brain region involved with reward processing and appetitive behaviors (Castro et al., 2015). Experiments differ in the use of microdrive connectors: the first one uses the Mill-Max connector, while the second one uses an Omnetics connector. They also differ in the rat strain (Wistar and Sprague-Dawley rats) and task used to assess the microdrive: one experiment involves an instrumental task pressing-lever to obtain food while the other one involves a licking task to obtain a liquid solution.

#### Subjects

Two male Wistar rats (280–300 g; Instituto de Fisiología Celular’s breeding colony) were housed in polyethylene cages. Rats were maintained on a 12 h light/dark cycle and fed standard laboratory rat chow in a restricted manner (18 g/day) until they reached 85% of their free-feeding weight. Rats had free access to water throughout the experiment. All manipulations and behavioral procedures were performed during the light phase. Animals were housed individually in a temperature-controlled environment (24°C) for at least 4 days before surgery. Animals were handled daily to diminish stress responses.

Five male Sprague-Dawley rats (300–350 g; CINVESTAV bioterium). Rats were housed in polyethylene cages and maintained on a 12 h light/dark cycle (lights on 0700; and lights off 1900). These rats were under a water restriction of 23 h per day, with 1 h of access to tap water in their home-cages following each recording session (see below). Such water restriction protocol is necessary for rats to maintain high levels of motivation for operant conditioning tasks (Hughes et al., 1994), without affecting the health of the rats (Rowland, 2007). Chow food diet (LabDiet, 5008) was available *ad libitum* in their home cages. All manipulations and behavioral procedures were performed during the light phase. Animals were housed individually in a temperature-controlled environment (21–24°C) for at least 4 days before surgery.

All procedures were approved by the Institutional Animal Care and Use Committee of the UNAM and CINVESTAV, in compliance with the National Ministry of Health guidelines for the care of laboratory animals.

#### Surgery

**FIGURE 6:**
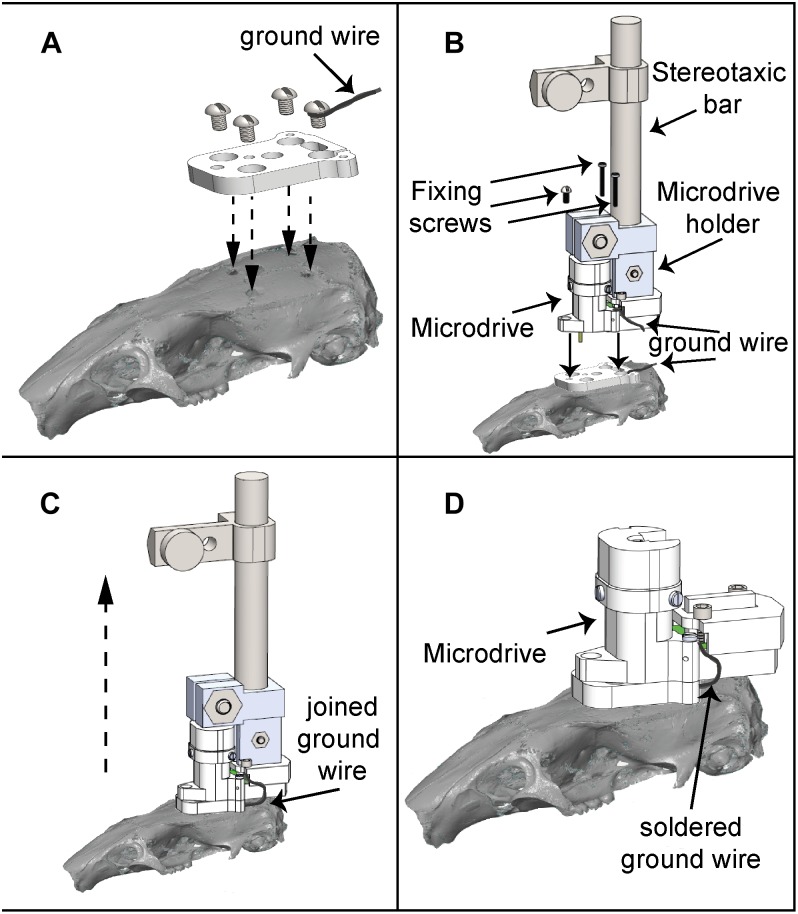
Microdrive implantation procedure. **(A)** The baseplate is located on the cranium of the rat according to the recording site and fixed using four screws. **(B)** The microdrive holder allows a smooth and precise mounting of the microdrive, which is fixed to the baseplate using three screws. **(C)** After the ground wire fixation, the microdrive is released from the holder and ready to use. **(D)** Microdrive firmly attached on the baseplate.

Figure 6 depicts the microdrive implantation procedure. Following behavioral training, Wistar rats were anesthetized using isoflurane, and Sprague-Dawley rats with an intraperitoneal injection of ketamine (90 mg/kg)/xylazine (8 mg/kg). The microdrive contained a 16-channel electrode bundle. For the five Sprague-Dawley rats the electrode bundle was implanted targeting the nucleus accumbens shell (NAcSh; +1.4 mm AP, -1 mm ML, and -7.5 mm DV, from bregma; Tellez et al., 2012), whereas Wistar rats aimed the NAcc (2.0 mm AP, +1.4 mm ML, and -6.0 mm DV, from bregma). Rats were positioned in a stereotaxic apparatus to perform a ∼2 × 2 mm craniotomy above the implantation site. Later, four additional Ø1.5 mm holes were made in the skull. The positions of these holes matched the holes in the baseplate where the microdrive was mounted. Then, the baseplate was fixed to the skull by screwing it through the holes. A drop of glue (cyanoacrylate, Krazy Kola Loka) was added to strengthen the fixation to the scalp. Finally, the reimplantable microdrive, positioned above the baseplate was lowered using a holder to introduce the electrode array in the brain. Once the microdrive was settled in, it was screwed to the baseplate. Following surgery, a triple antibiotic ointment was applied to the wound and enrofloxacin (45 mg/kg) was administered for three additional days. Rats were allowed to recover for 7–14 days before initiating recordings.

#### Behavior

Wistar rats were trained in standard operant chambers (Coulbourn Instruments, Allentown, PA, United States) located inside sound-attenuating boxes (MED Associates, Burlington, VT, United States) to press a bar to obtain food pellets (Bioserve, Inc., Frenchtown, NJ, United States) on a continuous reinforcement schedule. Recordings were performed in freely behaving rats or during lever pressing sessions.

Sprague-Dawley animals were recorded at the same time of the day between 2:00 and 3:00 p.m. Specifically, three rats were recorded while performing a brief-access test as described in Villavicencio et al. (2018). Briefly, taste stimuli consisted of reagent-grade sucrose (Sigma-Aldrich, Mexico) dissolved in deionized water. For the brief-access taste test, four semi-logarithmically spaced sucrose concentrations were used: 3, 5.8, 10.7, and 20 wt./vol. % (0.087, 0.168, 0.31, 0.58 M, respectively). For the brief-access test, in each trial, rats licked the spout to receive one randomly selected sweet concentration during a 5-s reward period. Moreover, the other two Sprague-Dawley rats performed an *ad libitum* freely licking sucrose test, in which only a sucrose solution of 10 wt./vol. % was available at all times (Tellez et al., 2012). Sweet tastants were prepared daily and used at room temperature. The behavioral box contained a V-shaped licking port where the spout was located (Med Associates, St. Albans, VT, United States). The licks given to the spout were recorded with a photobeam diode (Med Associates), and each lick delivered a 10-μL drop directly onto the rats’ tongue as described in Gutierrez et al. (2010).

#### Data Analysis

Custom-made Matlab (The MathWorks Inc., Natick, MA, United States) codes were used to analyze behavioral and single unit activity. To identify neurons whose activity was modulated while the rats licked for sucrose in both licking tasks (Figure 9D), the rhythmic licking behavior was first segmented into bouts. A lick bout was considered to be a train of contiguous licks with inter-lick intervals <500 ms (Spector et al., 1998; Tellez et al., 2012). Then, the neuronal firing rate was calculated during a baseline period encompassing -1 to -0.5 s before the onset of every lick bout and the firing rate during the lick bouts. Finally, to determine whether each neuron was modulated by licking or not, the firing rate in the baseline was compared with that in the licking period (lick bout) with a one-way ANOVA (α = 0.05). All neurons with a significant difference were classified as modulated by licking and the difference between the firing rate during the two periods was used to classify the direction of the modulation into Lick-Active (increases in firing rate) or Lick-Inactive (decreases). The proportion of Lick-Active and Lick-Inactive neurons found during the first and second implant was compared against that previously reported in Tellez et al. (2012), using the chi-squared test (α = 0.05; Figure 9E).

Furthermore, to compare the performance between two consecutive implants in the same animal, we performed the following statistical analyses. We counted the number of neurons recorded in each session using either a First or Second implant and compared among them with a Kruskal–Wallis test (α = 0.05; Figure 9B). Also, we counted the number of days each microdrive recorded unitary activity and compared between First and Second implants with a Kruskal–Wallis test (α = 0.05; Figure 9C). To control for the lower number of repetitions (implants), we modeled the number of recording days for either the First and Second implants by resampling. To achieve this, we bootstrap the data 10,000 times with replacement. Then, we calculated the confidence intervals (CI’s) centered at the 95% from the resulting distributions and looked if CI’s from the First and Second implants overlapped, to determine significant differences.

#### Multi-Channel Unit Recording

For the Wistar rats, individual neurons were recorded extracellularly using the present microdrive (with a Mill-Max connector), in freely behaving rats or while they pressed for food. Extracellular waveform signals exceeding a voltage threshold were amplified (gain × 100), digitized at 40 kHz using an OmniPlex Neural Data Acquisition system (Plexon, Dallas, TX, United States), and stored on a disk for further off-line analysis. Waveforms were recorded during 10 min periods of spontaneous activity. Single units were isolated using principal component analysis and template matching (Offline Sorter; Plexon, Dallas, TX, United States). We applied semi-automated (automated followed by manual correction) processing techniques to sort spikes from single units in clusters. Automated processing involved using a valley-seeking scan algorithm (Offline Sorter; Plexon, Dallas, TX, United States), one channel at a time, and then evaluated using sort quality metrics. For manual verification of automated clustering techniques, a cluster was considered to be generated from a single neuron if the cluster was distinct from clusters for other units in the principal component space. Also, the cluster had to exhibit a clear refractory period (>1 ms). Only stable clusters of single units during recording were considered for analysis. Timestamps of neural spiking and flags for the occurrence of bar-pressing were imported to NeuroExplorer (NEX Technologies, Littleton, MA, United States) for analysis. Long-term single-unit stability isolation was evaluated using Wavetracker (Plexon, Dallas, TX, United States) in which principal component space-cylinders were calculated from a 10 min segment of data spontaneously recorded session. Straight and overlapped cylinders or tubes suggest that the same single unit was recorded over different sessions (as in Herry et al., 2008).

For the Sprague-Dawley rats, extracellular single-unit activity from the NAcSh was recorded using a Multichannel Acquisition Processor (Plexon, Dallas, TX, United States). Specifically, voltage signals were sampled at 40 kHz and digitalized at 12-bit resolution. Single unit timestamps were extracted from the raw signal by using an online band-pass filter with a low cutoff of 154 Hz and high cutoff of 8.8 KHz. Only single neurons with action potentials having greater than 3:1 signal to noise ratio were analyzed. The action potentials were isolated online using voltage-time threshold windows and a three principal component contour template algorithm. Furthermore, for all recordings, off-line spike sorting (Plexon offline sorter) was performed, and only single units with stable waveforms across the entire session were included in the analysis (Gutierrez et al., 2010). As electrodes tips were positioned in the dorsal region of the NAcSh, we sampled neuronal responses across the dorsoventral axis by lowering down the electrodes every day. To this end, the lead screw driving the micro positioning system was turned one rotation before each experiment, pushing down the electrode bundle ∼250 μm. As the total displacement length of the reimplantable microdrive is 2.5 mm, after 10 days of recording (thus, traveling all the way down) the microdrive was replaced. For that purpose, rats were anesthetized and positioned in the stereotaxic frame. Once there, the microdrive was unscrewed and removed, leaving the baseplate fixed to the skull of the rat. A new microdrive was immediately reimplanted in the NAcSh using the same brain coordinates and screwed to the baseplate. During the following 3 days, rats had access to water in their home cages. Note that the reimplantable microdrives were lowered each day to test their capability to record single-unit activity all the way down and to replace the microdrives rapidly. However, of course, the experimenter can decide to lower and replace the microdrives, when considered necessary, depending on the intended duration of the experiment and the quality of the extracellular recordings.

#### Microdrive Placement and Electrode Location

To identify the location and integrity of the microdrive implant, a computed tomography (CT) was performed of the head of the rat using a Nikon CT, model XT H 225. Figure 9B is a representative X-ray image of the rat’s head with the microdrive implant in place. Upon completion of all experiments, rats were transcardially perfused with 0.9% saline solution followed by an overdose of pentobarbital (150 mg/kg, i.p.). Brains were extracted and fixed in a 30% sucrose/10% formalin solution (Sigma-Aldrich, St. Louis, MO, United States). Electrode placements were verified by cutting coronal sections 40–50 μm thick using a cryostat (Leica, CM1520), subsequently mounted on slides and stained for Nissl bodies with cresyl violet. Only rats with electrode locations within the borders of the target structure were included in the statistical analysis.

## Results

### Microdrive Assessment

To evaluate the implantation and reimplantation procedures of the microdrive, the time required for each procedure was measured. For the first case, the procedure consists in the surgery for attaching the baseplate by means of four screws, one of these with a silver ground wire soldered (Figure 6A); to place the microdrive at its final position, a customized microdrive holder was attached to the stereotaxic incisor bar; once the microdrive was on the baseplate, it was fixed by means of three fastener screws (Figure 6B); after this, the microdrive holder was released (Figure 6C); finally, the silver ground wire had to be soldered to the microdrive ground wire (Figure 6D).

For microdrive reimplantation, the procedure consists in releasing three fastener screws to remove the old microdrive (using the microdrive holder), cutting the ground wire in half (leaving at least 2 cm of ground wire from the ground screw attached to the skull to ground the EIB pin), cleaning the baseplate top and, after that, the procedure followed for implantation must be repeated (see Figure 6). We found that the average temperature on the ground screw after five soldering iterations (3 s per iteration) was 23.2°C (see Supplementary Figure S3). Even if soldering lasted as much as 10 s, we found that heating only reached 32°C, suggesting that heating due to re-soldering does not damage the underlying brain tissue.

**FIGURE 7:**
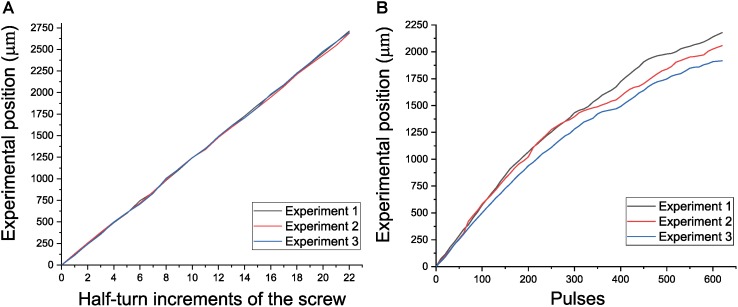
Performance of the electrode positioning system. **(A)** Experimental position versus half-turn increments of the screw. **(B)** Experimental position versus motor steps in open loop.

The total weight of the reimplantable microdrive, including the weight of the baseplate, is about 4.08 g when the nut-screw is used, and 4.15 g if the piezoelectric motor is implemented. In both configurations, its overall dimensions are 27.73 × 15.95 × 21.50 mm. The microdrive offers a travel range of 2.5 mm, which is a reasonable distance to cover most brain regions in a rat. To assess the movable mechanism proposed in the microdrive, two different characterizations were performed. The first one was related with assessing the smoothness of the displacements for demonstrating the reliability of the micropositioning system to record neural activity to a certain depth without lateral motions of the tip of the wire. The second one was focused on assessing the position capabilities of the microdrive. For both evaluations, an optical comparator NIKON Profile Projector V-16D with an ×20 magnification was employed, which is a precise instrument suitable for measuring different shapes of workpieces with a resolution of 0.1 μm. Both evaluations were performed in manual and open-loop motorized configurations. Once the microdrive was fixed on the profile projector stage, for the manual system, the screw was rotated 22 times in half-turn increments to generate forward and backward displacements with a theoretical value of 125 μm in each event. Three experiments were performed, and the experimental displacements were plotted versus the theoretical position (see Figure 7A). The mean displacements for the three experiments were 123, 122.1, and 122.5 μm, respectively. While the mechanism was displaced forward and backward, it was noticed that the electrodes have minimal, if any, lateral motion, which warranties a smooth displacement. The same procedure was performed using the automated microdrive with open-loop control. To generate the control signals, the NSD-2101 piezo motor driver by Austria Microsystems was employed. The actuator works by voltage pulses. To evaluate the displacement of the automated microdrive in an open-loop control, three experiments were performed. Each experiment was composed of 64 steps, and each step was programmed with 10 pulses, duration of 1 ms, and intervals of 1 ms. For this case, Figure 7B shows the experimental displacement as a function of the motor steps for the three experiments, thereby obtaining an average displacements of 35.145, 33.193, and 30.919 μm, respectively. As was observed in the first experiment, lateral deviations were not present.

### Long-Term Stable Neuronal Recordings in Freely Moving Rats

**FIGURE 8:**
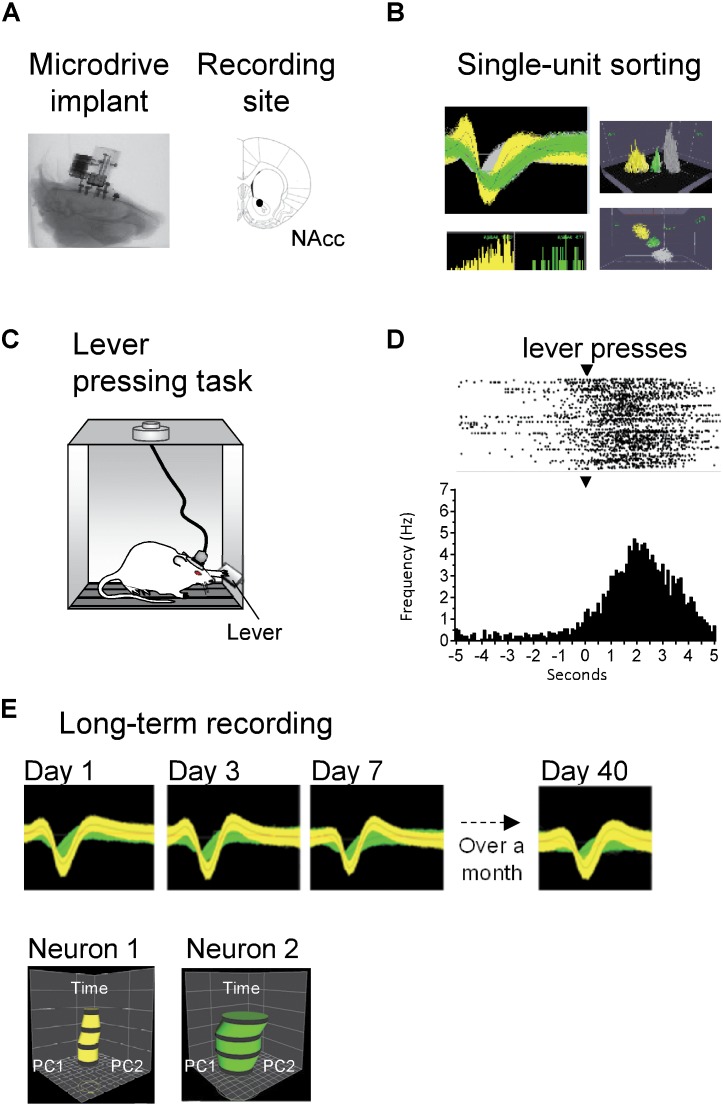
Long-term stable recordings of NAcc neurons in freely behaving rats. **(A)** Left: X-ray of the head of the rat showing location of the microdrive implant. Right: Histological reconstruction of the location of electrode tips in NAc of the ventral striatum. **(B)** Left top: Superimposed waveforms from two different units (green and yellow) recorded simultaneously in the same electrode and separated from noise (gray). Grid: 400 μV, 800 μs. Left bottom: Interspike interval histogram of the two units. Right: Offline sorting of two units using voltage-time threshold, principal component time-space surface (top) and 3D (bottom). **(C)** Freely behaving rats pressed a lever to obtain a food pellet, in a fixed ratio 1 reinforcement schedule. **(D)** Raster plot (top) and peri-event time histogram (bottom) for a neuron exhibiting an increase in firing rate after lever pressing (time = 0 s; the waveform of this neuron is shown in yellow in panel **B**). In the raster, a black tick indicates one action potential; the bin size was 100 ms. Arrowheads (▾) indicate the time of lever pressing (time = 0 s). **(E)** We tested the stability of recordings during spontaneous activity sessions in another freely behaving Wistar rat. Top: Waveforms of two separate neurons (yellow and green) recorded from the same single electrode for over a month (40 days). For visualization purposes, only waveforms in days 1, 3, 7, and 40 are shown. Bottom: Verification of long-term stable single-unit recordings using principal component space cylinders. A straight cylinder indicates that the same single unit was recorded in different sessions across time (days).

To assess the stability of the microdrive *in vivo*, we recorded NAcc neurons (Figure 8) in Wistar rats that were either in their home cage or during a behavioral task. Superimposed waveforms from two different units recorded simultaneously in the same electrode show them separated from noise. Offline sorting of two units using the voltage-time threshold, principal component time-space surface, interspike interval histogram, and 3D were used to verify they were separate units (Figure 8B). To validate the microdrive and evaluate its recording stability, freely behaving rats were trained in a lever-pressing task (Figure 8C). Spontaneous neural activity was recorded while rats were pressing a bar to obtain a food pellet. The response of one individual neuron is shown in the raster plot in Figure 8D. This neuron responded vigorously when pressing the lever to obtain food. This result is consistent with previous reports showing lever press response-related activity in NAcc (e.g., Morrison and Nicola, 2014). This neuron was recorded for three consecutive days (data not shown). These results indicate that the microdrive can be used for short-term (less than a week) recordings in rats performing a simple behavioral lever-press task. Further, in a different rat and to evaluate if the microdrive can be used for long-term recordings (over a month), we recorded spontaneous NAcc neuronal activity of two single neurons for 40 days. The response of these two individual neurons with a stable waveform for over 40 days is shown in Figure 8E. Quantitative verification of long-term stable single-unit recordings was performed using principal component space cylinders. A straight principal component space cylinder indicates that the same single unit was recorded in different sessions (days) across time. Taken together these results indicate that the microdrive is stable for short-term and, most importantly for long-term recordings in freely behaving rats. Yet, because of the limited dataset (one recording channel with two clearly isolatable units tracked for over a month), we acknowledge that implanting more rats with this microdrive would be necessary to evaluate quantitatively further the degree of long-term recording stability.

### Neuronal Recordings for the First and Second Microdrive Implant

**FIGURE 9:**
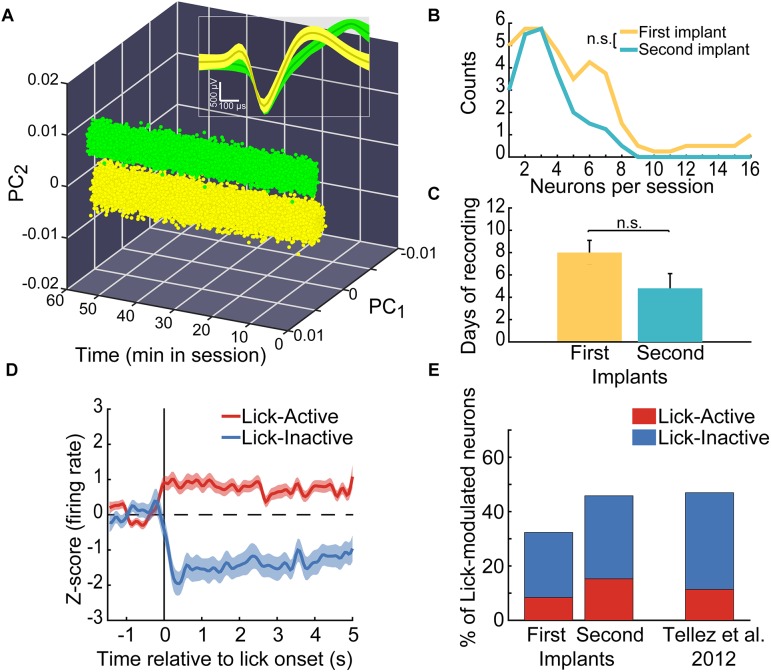
In the same animals, we recorded a similar proportion of lick-modulated NAcSh neurons in the first and second microdrive implant. **(A)** Action potentials (dots) of two single-neurons recorded in the same channel (yellow and green, respectively) within a 1 h freely licking behavioral session. Values are plotted in a three-dimensional space built with the first two principal components (PCs) of the voltage signals vs. time. Inset: Depicts the time-voltage window showing the mean ± SEM of action potential waveforms. These neurons were recorded from the “first” implant. **(B)** Histogram depicting the number of single neurons recorded in the first and second implant across sessions. **(C)** Number of daily sessions where single-unit activity was recorded in the first and second implant. **(D)** The population activity (normalized to z-score) of neurons that were either Lick-Active (red) or Lick-Inactive (blue) around the initiation of licking (vertical line at time = 0 s). The baseline level of neuronal activity (z-score = 0) is indicated by a horizontal dashed line and was taken from –1.5 to –0.5 s relative to the lick onset. **(E)** Histograms of the percentage of Lick-Active and Lick-Inactive neurons found in the NAcSh using the first and the second implant of the microdrive and the proportion of the lick modulated neurons reported in a study published elsewhere (Tellez et al., 2012). The proportions of neurons were not statistically different relative to data from Tellez et al. (2012) (chi-square test; Lick-Active neurons of the first and second implants vs. Tellez, χ^2^ = 0.004, and χ^2^ = 0.005, respectively; Lick-Inactive of the first and second implants vs. Tellez, χ^2^ = 0.017 and χ^2^ = 0.003, respectively; all *p’*s > 0.05).

Having characterized the stability of the microdrive, we then assessed if it was feasible to re-implant it without overtly affecting the functional activity of the targeted brain region. To do this, we performed extracellular single-unit recordings in five Sprague-Dawley rats performing a sucrose licking task. In total, the activity of 262 single neurons was recorded and analyzed to test the efficacy of our reimplantable microdrive. Figure 9 shows a representative example of two neurons recorded by the same tungsten wire. Note that the clusters of waveforms were stable and well separated in the principal component-time space, as well as in a voltage-time window (Inset) during the 1-h experiment, demonstrating once more that our microdrive can perform stable recordings in freely moving rats (in Supplementary Figure S2 can be found the raw data of online sorting of a single recording day).

Next, we characterized the quality of the recordings in two consecutive implants, performed in the same animal. The frequency to record single neurons between the First and Second implant was not statistically different (Kruskal–Wallis, χ^2^_(1,9)_ = 0.55, *p* = 0.45; Figure 9B; 4.7 and 3.2 neurons per session, respectively). Moreover, in average the activity recorded from the Second implant lasted roughly the same number of days than the First implant (Figure 9C; Kruskal–Wallis, χ^2^_(1,9)_ = 2.18, *p* = 0.14; *n* = 10 implants), suggesting that the process of degradation of the electrode’s signal was similar between both implants. To further validate this point, we resample with replacement the number of recorded days with signal. The averages of resampled data were 8.05 (CI = 1–7) and 5.33 (CI = 5–11) days with signal for the First and Second implants, respectively. Since confidence intervals did overlap, then it indicates no statistical difference, at a 95% confidence, exists between the First and Second re-implant. Nevertheless, we do acknowledge that in our second implant, we could not record more than eight simultaneous neurons in a single session (Figure 9B), further studies should confirm this trend. These data suggest that good-quality recordings can be obtained from a second implant in the same rat. Also, these results demonstrate that recordings in freely moving animals can be extended for at least one more implant (perhaps more), but this remains to be tested.

To further characterize if there is any reliable advantage in the yield of neurons recorded with our microdrive, we compared the yield obtained with our reimplantable microdrive against a permae against a permanently implanted movable electrode from our previously published study (Tellez et al., 2012) (see Table 1). In average, in the First implant, we recorded 36.8 neurons per animal, whereas with a permanently implanted electrode 38.3 (Mann–Whitney Rank *p =* n.s.), producing a yield of 4.7 vs. 5.7 neurons/session, respectively (*p* = n.s.). Thus, our microdrive results in a similar yield than other tungsten electrodes, at least, in the First implant. As noted above, the advantage of our drive mainly resides on the ability to be reimplanted (and to be reused). As can be appreciated in Table 1, we recorded, from the same rats, in average 16.2 new neurons in the Second implant (see Table 1; Second implant). Nevertheless, we acknowledge that not in all subjects the Second implant recorded more neurons than the First implant (see for example rat R02). Nonetheless, it is important to note that we also found cases (such as in subject R01) where the Second implant improved the yield of neurons, demonstrating the advantage of using our microdrive.

**Table 1:** Comparison of the yield of neurons obtained in First and Second implant, and against Tellez’s database with a permanently implanted electrode.

	First implant			Second implant		
Name rat	Sessions	Neurons	Neu/Sess	Sessions	Neurons	Neu/Sess
R01	6	6	1	6	22	3.6
R02	9	33	3.6	1	3	3
R03	11	71	6.4	7	25	3.5
R04	9	25	2.7	7	17	2.4
R05	5	49	9.8	4	14	3.5
Average	8	36.8	4.7	5	16.2	3.2

		**Permanent implantation**				
**Name rat**		**Sessions**		**Neurons**		**Neu/Sess**

Cooper		5		26		5.2
Floyd		8		59		7.3
Orec		3		10		3.3
Rhino		9		45		5
Silverio		14		147		10.5
Zero		5		15		9.4
Stan		1		4		4
Cua		1		1		1
Average		5.7		38.3		5.7


After demonstrating the recording capabilities of our microdrive, we evaluated whether the two consecutive implants lead to recording the same type of functional ensembles in the NAcSh. A considerable amount of evidence has consistently shown the existence of a small proportion of Lick-Active and a larger population of Lick-Inactive neurons in the NAcSh (Roitman et al., 2005; Krause et al., 2010; Tellez et al., 2012; Villavicencio et al., 2018), suggesting that these neuronal populations reflect the functional organization of the NAcSh. Thus, to address this issue, we first identified NAcSh neurons that were modulated during consummatory licking behavior. Figure 9D shows the populational activity of all NAcSh neurons that either increased their activity during licking (see red line) or suppressed it (blue line). Thus, the proportions of Lick-Active and Lick-Inactive neurons using our reimplantable microdrive was compared against the proportions reported in our previously published study (Tellez et al., 2012) (Figure 9E). For this analysis, the licking-modulated neurons from both, the brief-access taste test and the free-licking task, were pooled. No statistical differences were found in the percentage of active or inactive licking-modulated populations for the first or second implant in comparison with the data reported by Tellez et al. (2012) (chi-square test; all *p*’s > 0.05, *n* = 87 Lick modulated neurons from our microdrive vs. *n* = 124 from Tellez et al., 2012). This result suggests that the reimplanted microdrive can record the same functional ensembles as other standard electrodes without affecting the functional activity of the NAcSh. Altogether, the data obtained indicate that the microdrive is suitable for *in vivo* recordings involving simultaneous behavioral testing and, more importantly, that it can extend recordings for an extra period, without affecting neither their quality nor the functionality of the brain region targeted.

## Discussion

A novel microdrive design is presented here. This microdrive met the low cost, small size, and lightweight criteria, which are ideal features for experiments involving freely moving rodents. The assembly and implantation, as well as the re-implantations, resulted easy and quick. Also, the design presented here incorporates a mechanism based on two concentric cylinders that produce smooth displacements in both the manual and automated versions. Most important, it was demonstrated that the micropositioning system could reliably advance the electrode bundle in the space without lateral motions artifacts. One of the innovative aspects of the device is that is it potentially reusable. We have indeed been reusing parts from our microdrive by cleaning, re-plating and sterilizing the device (or by simply replacing the tungsten wires). This suggests that our microdrive allows for users to potentially re-use microdrives across animals, greatly decreasing cost and time of experiments. As a proof of concept, single unit recordings using the microdrives were tested in rats performing behavioral tasks or during spontaneous activity. We found a high-quality neuronal signal that was comparable between a first standard implant and a re-implant in the same subjects. Also, it was demonstrated that such a signal could last over a month. Thus, the microdrive design proposed here is an original and useful device to facilitate the collection of neuronal activity datasets, especially for experiments where time, training, and resources employed are critical.

### Micropositioning System

The microdrive presented incorporates a mechanism based on two concentric cylinders that produce smooth displacements; the mechanism is coupled to the lead screw by pressure, avoiding any backlash and system misaligning, which are the main drawbacks in mechanisms based on leadscrew and cannula guides (e.g., Fee and Leonardo, 2001; Venkateswaran et al., 2005; Otchy and Ölveczky, 2012; Jovalekic et al., 2017), in which the electrodes can be subject to mechanical drift and in consequence the loss of signal. The microdrive proposed exhibited good stability over several recording days. Another advantage of the microdrive presented is the possibility to choose between a manual or automated actuation. The use of the piezoelectric motor SQL-RV-1.8 allows replacing it with a nut and a screw with the same geometrical characteristics decreasing the microdrive’s cost. The mechanical characterization demonstrated that there were not lateral displacements along the displacement stroke. Related to the displacement characterization, for the manual configuration, a resolution of about 125 μm per half turn was obtained; but if the user requires a lower displacement, he/she only needs to decrease the rotation of the screw; for example, a quarter of a turn represents a displacement of about 60 μm. For the automatic configuration (open-loop control), it was observed that there was no similarity in the three experiments. Such a behavior is related to the actuator and the mechanism employed: ultrasonic PZT actuators usually do not have a constant behavior because their principle work is based on friction; on the other hand, the spring employed in the mechanism produces a non-constant preload force to the motor, which changes depending on the position in the microdrive stroke. Despite this drawback, using close control loop can improve the precision of the displacement, as demonstrated in Caballero-Ruiz et al. (2014), where a resolution of 1.3 μm with an average error of 2.2 μm was obtained with the prototype of the presented mechanism, making it a high-performance system.

### Manufacturing

The use of additive manufacturing technologies helps us to generate a microdrive housing with internal conduits to protect the microelectrodes form possible damages caused by the natural behavior of the animal; this is an important issue in extracellular recording experiments with rodents. Also, we incorporated a baseplate and a holder that permits the easy installation of the microdrive and the possibility of reimplanting a new one in case of loss of signal. This represents an advantage when trained animals are used for the experiment. The microdrive presented can displace a bundle of 16 microelectrodes, which makes it impossible to determine the specific location of each of them, individually. It is necessary to work on a proposal that allows moving a microelectrode array with a specific distance between the electrodes to avoid the possible crosstalk phenomena and to know the specific location of each electrode. The recording experiments were performed in the NAc of the ventral striatum, but the design can be easily modified to target other brain regions. The additive manufactured baseplate allows for a flexible recording location, located between AP ± 3.5 mm and ML ± 2.5 mm; with some minimal modifications in the 3D model, it is possible to achieve more distant regions; however, not for all brain regions, for example, this design cannot offer recordings in very lateral areas such as in the posterior insular cortex (ICp); therefore, designing a universal baseplate that can be used on any region of the brain can be considered. The final weight and dimensions of the proposed microdrive are appropriate for extracellular recording in rats. For smaller rodent species, such as mice, it will be necessary to miniaturize its components and redesign the baseplate and the microdrive. Another caveat of our microdrive is that all electrodes are moved in a bundle rather than an independent placement of each electrode individually. Nevertheless, this limitation allows us to reduced device weight and, for a future version, it could save space to introduce a second driver to record two simultaneous brain regions.

### Gold Plating of Electrodes

Sixteen tungsten wires with 35 μm in diameter were gold-plated in order to reduce the impedance to 70 kΩ. However, the user can flexibly choose electrodes with other diameters and materials. It has been demonstrated that tungsten is a good choice of material for chronic implants since it is a stable and inert metal (Freire et al., 2011), and it has been successfully used in single unit recordings (Gutierrez et al., 2010). This diameter is stiff enough to maintain stability and rigidity at lengths <7 mm. We note that larger diameters >50 μm are not optimal for isolating single unit activity. Furthermore, in Patrick et al. (2011), it was demonstrated gold-plated in tungsten electrodes. Additionally, Prasad et al. (2014) reported that with tungsten electrodes, the best yield is for the impedance range 40–150 kΩ. Further studies should analyze different materials and diameters in order to characterize the best option of electrodes and impedance range.

### Datasets Collection

The main advantage of using the microdrive is, of course, reducing the number of experimental subjects, which is a fundamental ethical issue as expressed by Institutional Animal Care and Use Committees (Fitts, 2011). We believe that our microdrive will be especially valuable for preparations for which extensive behavioral training is needed (Erlich et al., 2011; Jaramillo and Zador, 2014; Liu et al., 2014; Fonseca et al., 2018); or in animal models for chronic diseases that take months to develop, such as diet-induced obesity (Benedikz et al., 2009; Avena, 2012). We expect our reimplantable microdrive to become a critical implementation for extracellular recordings that facilitate the collection of larger neuronal activity databases over long periods.

In sum, here we presented the design of a novel microdrive that resulted in stable recordings over days to a month, and more important, when necessary it could be reimplanted, in the same animal, with the intent to extend the half-life of extracellular recordings. Altogether, the structural and functional features of the present microdrive make it ideal to further study the neuronal correlates of behaviors, in health and disease, from perception to decision making that requires stable recordings across several weeks and give the opportunity to reuse it and reimplant it if there is an error during surgery.

## Data Availability

The datasets generated for this study are available on request to the corresponding author.

## Author Contributions

LR-H, FS-B, RG, and AC-R designed the research. LP-C, LR-L, MV, MM, and EI-H performed the research. AC-R, LR-H, RG, and FS-B analyzed the data. LR-H, MV, FS-B, RG, LP-C, and AC-R wrote the manuscript.

## Conflict of Interest Statement

The authors declare that the research was conducted in the absence of any commercial or financial relationships that could be construed as a potential conflict of interest.

## Supplementary Material

Figure S1 Distance from baseplate to end of electrode bundle (7 mm). **(A)** Length of electrodes without the baseplate. **(B)** Same as in **(A)** but with the baseplate in place.

Figure S2 Single-unit activity recorded from the reimplantable microdrive. Screenshot of the acquisition system’s user interface software showing single-unit activity recorded during an ongoing experiment (online sorting). At the right, it is seen different time-voltage windows corresponding to different channels from where single-unit activity was recorded. Yellow and green traces distinguish different neuronal signals recorded in a single channel. At left side of the screenshot, it is shown an expansion of the channel “4” seen at the left (see window framed by a magenta rectangle).

Figure S3 Soldering process and temperature measurement. **(A)** Temperature measured with an infrared thermometer before soldering. **(B)** The ground wires have been joined. **(C)** The ground wires have been soldered and an average temperature of 23.2°C has been obtained.

Table S1 NanoZ stepped protocol used to perform the electroplating in the electrodes. This protocol was applied to each channel. The interval is the time between bi-phasic pulses within a run. Runs are the maximum number of attempts per step to reach a target impedance. n/a, not applicable.

Table S2 Costs for microdrive parts. For the automated microdrive, the total cost of the parts is 280,2 USD, while for the manual configuration is 155,2 USD. Both estimations were made considering the cost of the electronic interface board (101.65 USD), which is reusable. Without the cost of the electronic interface board, the total cost of each microdrive is 53,55 USD.

DATA SHEET S1 STL files and G code for manufacturing the parts of the microdrive.
