# Supplementary figures and images for: Reimplantable Microdrive for Long-Term Chronic Extracellular Recordings in Freely Moving Rats
Source: Front Neurosci. 2019 Feb 21;13:128. doi: 10.3389/fnins.2019.00128 (PMC6393392; doi:10.3389/fnins.2019.00128)

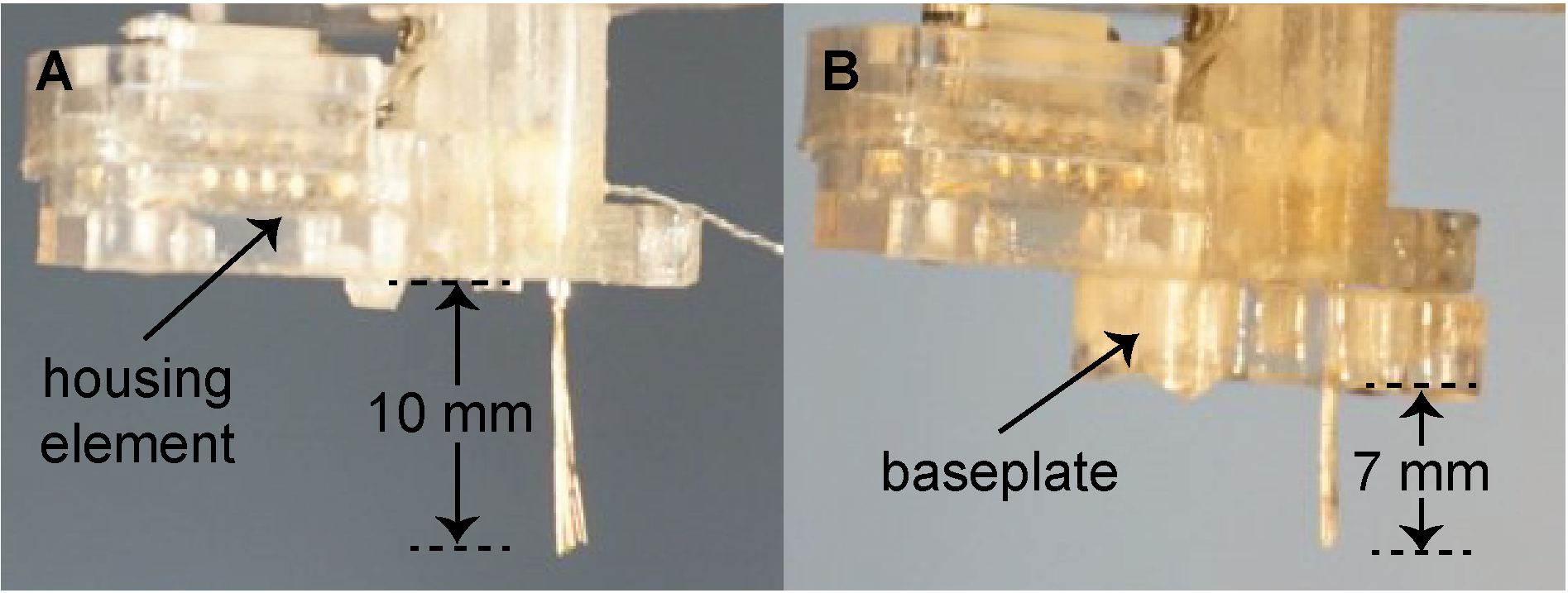

Supplement: Figure S1 — Distance from baseplate to end of electrode bundle (7 mm). (A) Length of electrodes without the baseplate. (B) Same as in (A) but with the baseplate in place. [file Image_1.TIF]

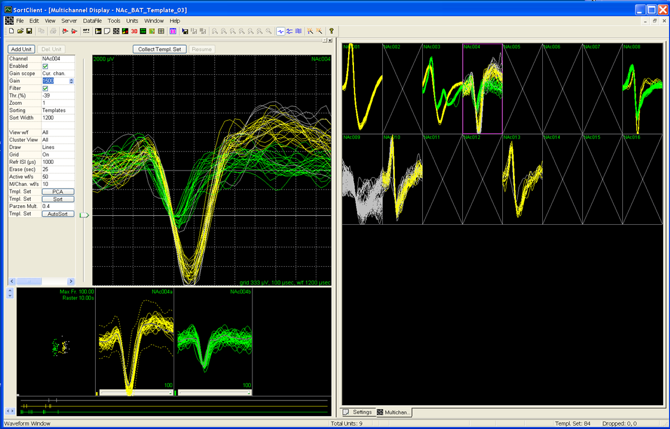

Supplement: Figure S2 — Single-unit activity recorded from the reimplantable microdrive. Screenshot of the acquisition system’s user interface software showing single-unit activity recorded during an ongoing experiment (online sorting). At the right, it is seen different time-voltage windows corresponding to different channels from where single-unit activity was recorded. Yellow and green traces distinguish different neuronal signals recorded in a single channel. At left side of the screenshot, it is shown an expansion of the channel “4” seen at the left (see window framed by a magenta rectangle). [file Image_2.TIFF]

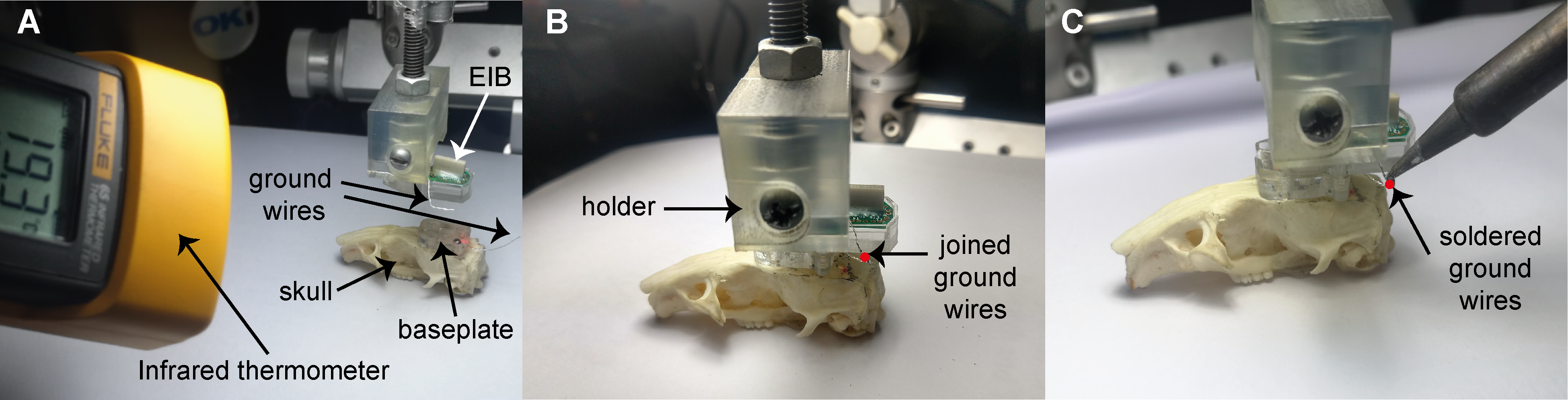

Supplement: Figure S3 — Soldering process and temperature measurement. (A) Temperature measured with an infrared thermometer before soldering. (B) The ground wires have been joined. (C) The ground wires have been soldered and an average temperature of 23.2°C has been obtained. [file Image_3.TIF]
