# Supplementary material for: Reimplantable Microdrive for Long-Term Chronic Extracellular Recordings in Freely Moving Rats
Source: Front Neurosci. 2019 Feb 21;13:128. doi: 10.3389/fnins.2019.00128 (PMC6393392; doi:10.3389/fnins.2019.00128)
Supplement: Table S1 — NanoZ stepped protocol used to perform the electroplating in the electrodes. This protocol was applied to each channel. The interval is the time between bi-phasic pulses within a run. Runs are the maximum number of attempts per step to reach a target impedance. n/a, not applicable. [file Table_1.DOCX]

| Step | Current (𝜇A) | Target (MΩ) | Interval (s) | Runs |
| --- | --- | --- | --- | --- |
| Cleaning | 0,1 | n/a | 1 | 1 |
| 1 | -0,05 | 2 | 15 | 3 |
| 2 | -0,05 | 1.5 | 10 | 3 |
| 3 | -0,05 | 0.5 | 5 | 3 |
| 4 | -0,05 | 0.25 | 3 | 3 |
| 5 | -0,05 | 0.1 | 2 | 3 |
| 6 | -0,05 | 0.07 | 2 | 3 |

**Supplementary Table 1. NanoZ stepped protocol used to perform the electroplating in the electrodes.** This protocol was applied to each channel. The interval is the time between bi-phasic pulses within a run. Runs are the maximum number of attempts per step to reach a target impedance. n/a, not applicable.
