# Supplementary material for: Reimplantable Microdrive for Long-Term Chronic Extracellular Recordings in Freely Moving Rats
Source: Front Neurosci. 2019 Feb 21;13:128. doi: 10.3389/fnins.2019.00128 (PMC6393392; doi:10.3389/fnins.2019.00128)
Supplement: Table S2 — Costs for microdrive parts. For the automated microdrive, the total cost of the parts is 280,2 USD, while for the manual configuration is 155,2 USD. Both estimations were made considering the cost of the electronic interface board (101.65 USD), which is reusable. Without the cost of the electronic interface board, the total cost of each microdrive is 53,55 USD. [file Table_2.DOCX]

| ID | Component | Unitary price (USD) | For all units |
| --- | --- | --- | --- |
| 1 | Piston | 10 | 10 |
| 2 | Bushing | 10 | 10 |
| 3 | Compression spring | 0,3042 | 0,30 |
| 4 | Polyimide tube | 0,67 | 0,67 |
| 5 | Cannula | 0,03 | 0,03 |
| 6 | Magnet | 0,086 | 0,09 |
| 7 | Hall-effect sensor | 2,56 | 2,56 |
| 8 | Nut | 0,5 | 0,5 |
| 9 | 10 mm screw | 0,85 | 0,85 |
| 10 | Piezoelectric actuator | 125 | 125 |
| 11 | Baseplate | 2 | 2 |
| 12 | Housing element | 5 | 5 |
| 13 | EIB cover | 2 | 2 |
| 14 | Electrodes cover | 2 | 2 |
| 15 | 3 mm fixing screws (x4) | 0,95 | 3,8 |
| 16 | 8 mm fixing screws (x2) | 0,72 | 1,44 |
| 17 | Anterior fixing screw | 0,05 | 0,05 |
| 18 | Skull screws (x4) | 0,05 | 0,2 |
| 19 | Electrodes (x17) | 0,45 | 7,65 |
| 20 | Electronic interface board | 101,65 | 101,65 |
| 21 | Large EIB pins | 0,214 | 3,64 |
| 22 | Silver ground wire | 0,768 | 0,77 |

**Supplementary Table 2. Costs for microdrive parts.** For the automated microdrive, the total cost of the parts is 280,2 USD, while for the manual configuration is 155,2 USD. Both estimations were made considering the cost of the electronic interface board (101.65 USD), which is reusable. Without the cost of the Electronic interface board, the total cost of each microdrive is 53,55 USD.
